# TNFAIP3 Facilitates Degradation of Microbial Antigen SEB in Enterocytes

**DOI:** 10.1371/journal.pone.0045941

**Published:** 2012-09-24

**Authors:** Chi Chen, Gui Yang, Xiao-Rui Geng, Xingpeng Wang, Zhanju Liu, Ping-Chang Yang

**Affiliations:** 1 Department of Gastroenterology, the Shanghai Tenth People’s Hospital, Tongji University, Shanghai, China; 2 Department of Pathology & Molecular Medicine, McMaster University, Hamilton, Ontario, Canada; Rush University Medical Center, United States of America

## Abstract

**Background and Aims:**

The enterocytes have the potential to absorb noxious substances, such as microbial products, from the gut lumen. How the enterocytes process the substances to harmless materials is not fully understood. This study aims to elucidate the role of ubiquitin E3 ligase TNFAIP3 (TNFAIP3) in facilitating the degradation of endocytic microbial products in enterocytes.

**Methods:**

Human intestinal epithelial cell line, HT-29 cells, was cultured to monolayers using as an *in vitro* model to observe the endocytosis and degradation of microbial products, Staphylococcal enterotoxin B (SEB) in epithelial cells. The RNA interference was employed to knock down the TNFAIP3 gene in HT-29 cells to observe the role of TNFAIP3 in the degradation of endocytic SEB. The role of TNFAIP3 in facilitating the endosome/lysosome fusion was observed by immunocytochemistry.

**Results:**

Upon the absorption of SEB, the expression of TNFAIP3 was increased in HT-29 cells. Silencing the TNFAIP3 gene in HT-29 cells resulted in a large quantity of SEB to be transported across the HT-29 monolayers to the transwell basal chambers; the transportation was via the intracellular pathway. TNFAIP3 was required in the fusion of SEB-carrying endosomes and lysosomes.

**Conclusions:**

TNFAIP3 plays a critical role in the degradation of endocytic SEB in enterocytes.

## Introduction

On the surface of the gut lumen, there is a single layer of epithelial cells that forms the gut epithelial barrier together with the tight junctions around the top of each epithelial cell. The gut epithelial barrier physically separates the intestinal tissue from the harsh environment of the gut lumen [1]. Another function of the gut epithelial cells is absorbing nutrients from the gut lumen. Although most proteins can be degraded by the proteases in the gut lumen before they are absorbed, still some peptides with antigenicity or small protein molecules are remained that may be absorbed by gut epithelial cells [2]. Theoretically, the amounts of peptides or protein molecules with sufficient antigenicity should be minimized to certain extents with which the body immune systems can tolerate without causing pathogenic response before they are delivered into the subepithelial region. Thus, it is expected that gut epithelial cells can degrade most of the absorbed peptides or proteins. Yet, the mechanism by which gut epithelial cells digest the absorbed substance is not fully understood.

The endocytosis is the basic mechanism by which gut epithelial cells absorb nutrients from the gut lumen. The absorbed molecules can be wrapped by the plasma membrane to form the endosomes; the latter then fuses with lysosomes [3]. There are a number of enzymes in lysosomes that can degrade the absorbed proteins and peptides. It seems that the fusion of endosome and lysosome is a key step to degrade the absorbed proteins. Several molecules are associated with the fusion of the endosome and lysosome such as Rab family [4], the homotypic fusion and vacuolar protein sorting (HOPS) complex [5] and soluble N-ethylmaleimide-sensitive factor-attachment protein receptor [6].

Recent reports indicate that the ubiquitin TNFAIP3 is associated with the tethering of endosome and lysosome [7]. TNFAIP3 is a ubiquitin E3 ligase in the cytosole that plays a critical role in the innate immunity and adaptive immunity, and is an important molecule in the maintenance of the homeostasis in the body [8]. Lacking TNFAIP3 in the gut epithelial cells results in severe gut inflammation [8]. TNFAIP3 is involved in the degradation of absorbed microbial products such as flagellin and lipopolysaccharide [9], [10]. Thus, we hypothesize that the expression of TNFAIP3 by gut epithelial cells upon the absorption of microbial products is a protective mechanism by which the gut epithelial cells degrade the absorbed proteins to minimize the amounts of noxious materials to be transported to the subepithelial region. In this study, we chose the Staphylococcal enterotoxin B (SEB), a well-characterized microbial product [11], in our experimental system. The results showed that upon exposure to SEB, the expression of TNFAIP3 was increased in the gut epithelial cells that facilitated the fusion of endosome and lysosome, and promoted the degradation of SEB in the enterocytes.

## Materials and Methods

### Reagents

Antibodies of TNFAIP3 shRNA, IgE, CD23, SEB, TNFAIP3, EEA1, LAMP2 and fluorescence labeled second antibodies were purchased from Santa Cruz Biotech (Shanghai, China). Reagents for qRT-PCR were purchased from Invitrogen (Shanghai, China). Immunoprecipitation reagents and SEB were purchased from Sigma Aldrich (Shanghai, China). Cell lines of T84, HT-29 and Caco-2 were purchased from ATCC (Manassas, VA).

### Cell Culture

T84 cells, HT-29 cells and Caco-2 cells were cultured in Dulbecco’s modified Eagle’s medium (DMEM) medium (Gibco) supplemented with 10% fetal bovine serum (FBS) and cultured at 37°C in a humidified incubator under an atmosphere of 95% O_2_ and 5% CO_2_.

### Measurement of Transepithelial Resistance (TER)

The cells were grown on Transwell® permeable supports (Corning). The TER was recorded using a Millicell-ERS Electrical Resistance System (Millipore) and presented as ohms·cm^2^. The baseline TER of the polycarbonate membrane of transwell (∼30 ohms·cm^2^) was subtracted from all readings.

### SEB Flux

HT-29 cells were cultured in Transwells to confluence. SEB was added to the apical chambers at a concentration of 10 µg/ml. Medium samples were taken from the basal chambers at 8 h, 16 h and 24 h respectively. The levels of SEB in the samples were determined by Enzyme-linked immunoassay (ELISA).

### ELISA

The contents of SEB and TNFAIP3 in samples were measured by ELISA. The sample protein (20 µg/ml; 0.1 ml/well), or the standard protein with graded dilutions, or bovine serum albumin (BSA, using as an irrelevant control antigen), was added to each well and incubated at 4°C overnight. The plates were blocked by 5% non-fat dry milk for 1 h. The primary antibodies (10 ng/ml) were added and incubated for 1 h that was followed by adding HRP-labeled secondary antibodies (5 ng/ml) and incubated for 1 h (Washing with TBS for 3 times between steps). TMB (3,3′,5,5′-tetramethylbenzidine) solution 0.1 ml was added to each well, incubated for 15–30 min, 25 µl of stopping solution (2M H_2_SO_4_) was added to each well to stop the reaction. The optical density (OD) of the wells was read at 450 nm with a microplate reader (BioTek, Shanghai, China). The OD value of the control wells was subtracted from each sample well or standard well. The contents of SEB and TNFAIP3 were calculated against the standard curve.

### Sensitization of Enterocyte Cell Line with SEB-specific IgE

HT-29 cells (5×10^5^/ml) were sensitized with anti-SEB IgE (100 ng/ml) and incubated overnight. The cells were then washed with PBS and subjected to further experiments.

### Immunocytochemistry

The procedures of immune staining were referred to our previous reports with minor modifications [12]. Briefly, cells were placed in eppendorf tubes, fixed with 2% paraformaldehyde for 2 h and blocked by 1%BSA for 30 min. The cells were incubated with the primary antibodies (0.5–1 µg/ml) at 4°C overnight that was followed by adding the fluorescence labeled secondary antibodies. Washing with PBS was performed after each step. The cells were smeared on a slide, covered with a cover slip and observed under a confocal microscope with the ×630 objective. When the positive staining was observed, the image was further enlarged with the built-in enlarge feature of the software; the fine structure of the positive staining was observed and photographed when appropriate. The samples were coded; the observers were not aware of the code to avoid the observer bias.

### Purification of SEB-specific IgE

By screening with ELISA, the serum with SEB-specific IgE contents greater than 30 ng/ml was collected from 3 patients with food allergy and 3 patients with allergic rhinitis at our hospital. The use of human sample in the study was approved by the Human Research Ethic Committee at Tongji University. SEB (10 µg/ml) was loaded onto the SDS PAGE gel and electrically transferred onto nitrocellulose membranes (similar to the procedures of Western blotting). The SEB-containing serum was added to the membrane and incubated in a humidified box for 30 min at room temperature. The bound antibodies were eluted with glycine buffer (pH 2.7), vortexed for 5 min. The eluted antibody was neutralized by adding 1/10 the volume of 2M Tris (pH 8.0). The contents of SEB-specific IgE were determined by ELISA.

### Real Time RT-PCR (qRT-PCR)

Total RNA was isolated from HT-29 cells using Trizol reagent following the manufacturer's instruction. One microgram of RNA was reverse-transcribed to obtain complementary DNA (cDNA). The expression of TNFAIP3 mRNA was assessed by qRT-PCR using primers of forward, cttgtggcgctgaaaacgaa, reverse, ccactgtccttcagggtcac (NCBI: NM_006290.2; for human gene); or the primers for mice: forward, tgcaatgaagtgcaggagtc; reverse, tgggctctgctgtagtcctt (NCBI: NM_001166402.1). Each reaction mix contained 2 µL of 10-fold diluted cDNA, 23 µL premix, 1 µL forward primer (10 µmol/L), 1 µL reverse primer (10 µmol/L), 8.5 µL RNase-free water, and 12.5 µL Power SYBR green PCR Master Mix. The PCR was performed using a Bio-Rad Thermal Cycler (Bio-Rad, Shanghai, China). The levels of mRNA were normalized to the percentage of the housekeeping gene β-actin.

### Immunoprecipitation Assay

HT-29 cell lysates (0.2 ml) were incubated for 2 h with anti-CD23 antibody (or isotype IgG using as a control) at 4°C; protein G-Sepharose was added to capture the resulted immune complexes. After centrifugation, the pellets were washed 3 times using sodium bicarbonate buffer. The samples were normalized to equal amounts of protein, separated by 10% SDS-PAGE, transferred onto nitrocellulose membrane, followed by probing with antibodies of CD23, IgE and SEB respectively. Detection was performed by incubation with HRP-conjugated secondary antibodies. Immunoreactive blots were visualized by enhanced chemiluminescence.

### Flow Cytometry

Single HT-29 cells were prepared with trypsin-EDTA and subjected with the surface staining with primary antibodies of CD23, IgE and SEB for 30 min on ice. After washing, the cells were incubated with the fluorescence labeled secondary antibodies for 30 min on ice and analyzed by flow cytometry. The gating technique was used in analysis of the data. The CD23^+^ cells were gated first; the IgE^+^ cells were detected from the gated CD23^+^ cell population; then, SEB^+^ cells were counted from the gated IgE^+^ cells.

### RNA Interference (RNAi)

HT-29 cells were transduced with shRNA of TNFAIP3 carried by lentiviral vectors following the manufacturer’s instruction. Control shRNA was introduced into the control cells.

### Animal Study

BALB/c mice (6–8 week old) were purchased from Charles River Laboratories (Saint-Constant, QC, Canada) and maintained in a pathogen-free environment. The mice were sensitized to SEB with alum as an immune adjuvant following the procedures we reported previously [13]. The animal study was approved by the Animal Care Committee at McMaster University.

### Statistical Analysis

The data were expressed as mean ± SD. All statistical tests were analyzed with the two-sided Student's t-test between two groups or ANOVA in more than two groups; p<0.05 was defined as the statistical significant criteria.

## Results

### SEB Binds IgE/CD23 Complexes on the Surface of Enterocytes

**Figure 1:**
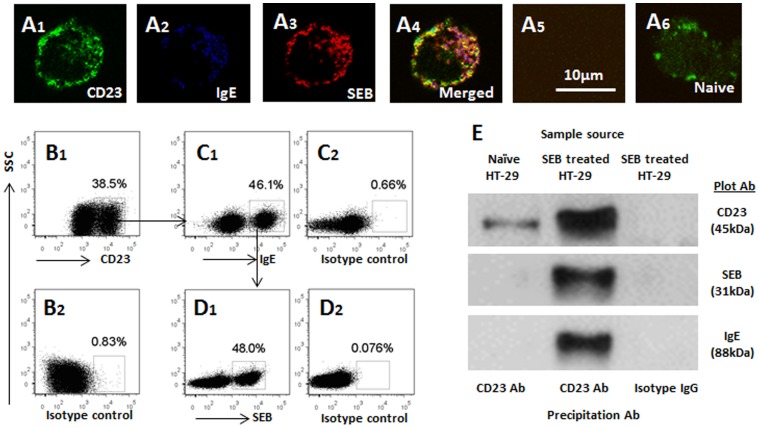
CD23 mediates SEB absorption in enterocytes. A, the representative confocal images show immune positive staining in HT-29 cells, which were exposed to SEB in culture (A1–A4) for 30 min. A5 is an isotype control. A6 is from naïve HT-29 cells. B-D, the representative flow cytometry dot plots show the surface staining of CD23 (B), IgE (C) and SEB (D). E, immunoprecipitation assay results show an immune complex of CD23/IgE/SEB in HT-29 cells. The data represent 3 separate experiments.

Published data indicate that enterocytes express CD23; CD23 is the low affinity receptor of IgE. To see if SEB-specific IgE could bind the CD23 to form immune complexes on the surface of enterocytes, enterocytes were cultured in the presence of SEB-specific IgE for 30 min at 37°C. The cells were collected and observed by immunocytochemistry. The confocal images in Fig.1A showed that a SEB/IgE/CD23 complex was localized on the surface of enterocytes. To confirm the results, the cells were analyzed by flow cytometry with the surface staining approach. The results showed CD23 was detected in 38.5±5.6% enterocytes (Fig.1B). Among the CD23^+^ enterocytes, 46.1±5.9% enterocytes showed IgE positive (Fig.1C) and 48.0±5.6% IgE^+^ enterocytes were also SEB^+^ (Fig.1D). In addition, total proteins were extracted from a portion of the enterocytes and analyzed by immunoprecipitation assay. The results showed a complex of SEB/IgE/CD23 was formed in the enterocytes (Fig.1E). The results indicate that enterocytes expresses CD23; SEB-specific IgE binds to the CD23 to form a complex; the latter can be bound by SEB.

### CD23 Facilitates SEB Internalization into Enterocytes

**Figure 2:**
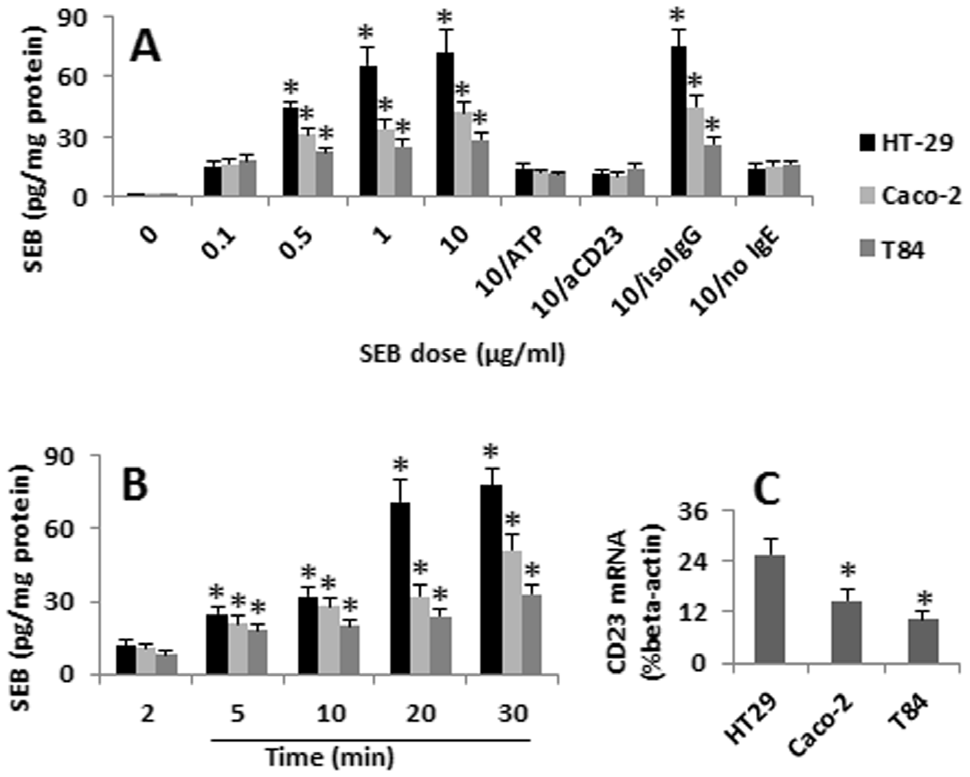
Enterocytes absorb SEB. Enterocytes (HT-29, Caco-2 and T84) were primed with SEB-specific IgE; after washing, were cultured in the presence of SEB. A, the bars indicate the levels of SEB in the extracted protein of enterocytes after exposure to SEB at graded doses in culture. B, the bars indicate the levels of SEB in the extracted protein of enterocytes after exposure to SEB at 1 µg/ml for 2–30 min (or 24 h). ATP (aCD23; isoIgG): The enterocytes were pretreated with ATP to remove the CD23 from the surface of the cells (or treated with neutralizing anti-CD23 antibody; or isotype IgG). no IgE: The enterocytes were not primed by specific IgE. The data were presented as mean ± SD. *, p<0.01, compared with the dose “0.1″ group (A) or time point “2 min” (B). C, the bars indicate the CD23 mRNA levels in the enterocytes (assessed by qRT-PCR). The data represent 3 separate experiments.

The confocal images in Fig.1 also showed some immune positive staining of SEB, IgE and CD23 was localized inside of the enterocytes. The fact indicates that the SEB/IgE/CD23 complexes were internalized into the enterocytes. We next performed ELISA to quantify the amounts of SEB in the enterocytes. As shown by Fig.2, the SEB levels in the enterocytes were in a time- and SEB dose (in culture media)-dependent manner during 0–30 min; however, extending the incubation time to 24 h did not further increase the absorption of SEB. To confirm the role of CD23 in the absorption of SEB, in separate experiments, the enterocytes were pretreated with ATP to remove CD23 from the cell surface [14], and exposed to SEB-specific IgE and then to SEB. Indeed, the absorption of SEB by enterocytes was abolished. Similar to the results by using ATP, pretreating the cells with neutralizing anti-CD23 antibody also abolished the increase of the absorption of SEB by the enterocytes (Fig.2A). To confirm the role of the IgE/CD23 complex in the absorption of SEB by the enterocytes, in a separate experiment, we exposed naïve enterocytes to SEB in the culture. Indeed, the contents of SEB in the enterocytes were significantly less than those primed with IgE (Fig.2A). We tested three enterocyte cell lines. The amounts of absorbed SEB were significantly more in HT-29 cells than other two cell lines. To see if the difference was caused by the expression of CD23 at different levels, we analysed the expression of CD23 in the three enterocyte cell lines. The qRT-PCR results showed that the expression of CD23 was indeed significantly higher in HT-29 cells than that in Caco-2 cells and T84 cells (Fig.2D).

### SEB Increases TNFAIP3 Levels in Enterocytes

**Figure 3:**
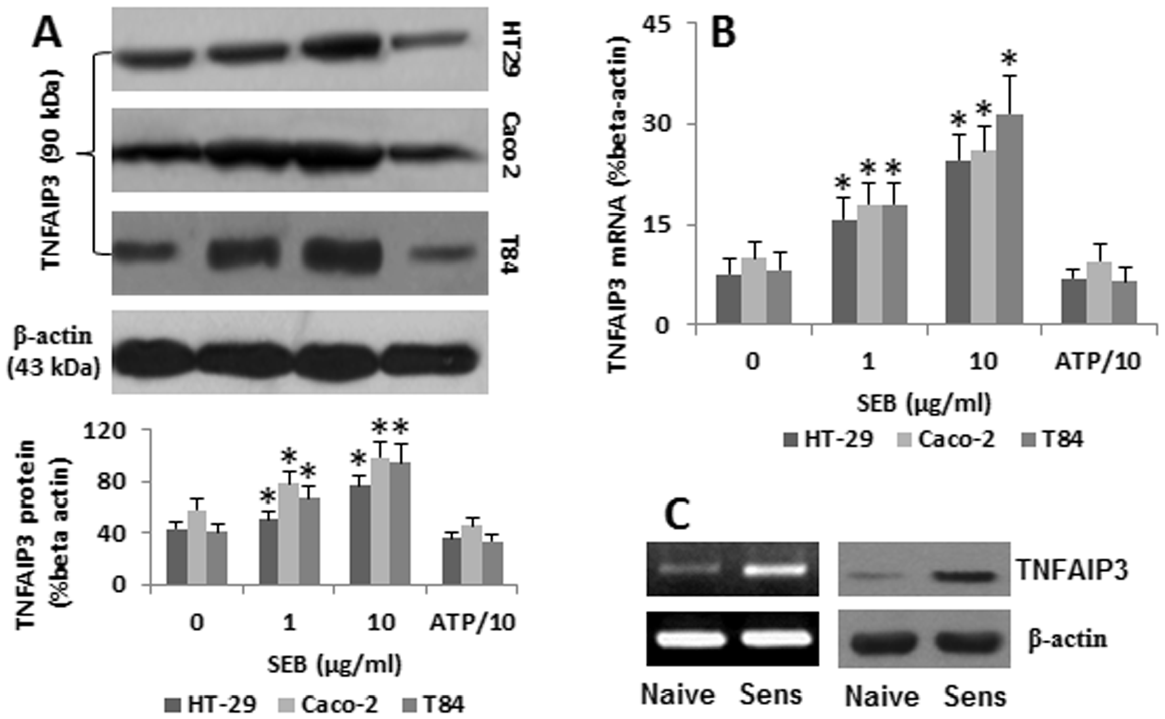
SEB increases TNFAIP3 expression in enterocytes. SEB-specific IgE-primed enterocytes were cultured in the presence of SEB for 48 h. The cells were processed to assess the TNFAIP3 expression. A, the immune blots indicate the TNFAIP3 protein levels in the enterocytes. The bars below the blots indicate the integrated density of the TNFAIP3 immune blots. B, the bars indicate the TNFAIP3 mRNA levels (by qRT-PCR). The data in bar graphs were presented as mean ± SD. *, p<0.01, compared to the dose “0″ group. C, jejunal epithelial tissue was taken from naïve mice and mice sensitized to SEB (Sens). The mRNA and protein bands show the expression of TNFAIP3. The data represent 3 separate experiments.

SEB is a superantigen that can directly modulate the T cell’s activities [15]; but solely exposing to SEB does not alter the enterocyte’s properties [16]. The fact implies that the absorption of SEB may trigger a protective action in enterocytes. Previous studies indicate that TNFAIP3 expression can be evoked in target cells after exposure to microbial products [9]; TNFAIP3 plays a role in the endocytic cargo degradation in the cells [17]; we thus assessed the expression of TNFAIP3 in the SEB-specific IgE-primed enterocytes after exposure to SEB. The data in Fig.3 showed that the exposure to SEB increased the expression of TNFAIP3 in enterocytes in a SEB dose-dependent manner. The removal of CD23 from enterocytes abolished the increase in TNFAIP3 (Fig.3), which indicates that the increase in TNFAIP3 is occurring after the absorption of SEB. To see if exposure to SEB could increase the expression of TNFAIP3 in the intestinal epithelial cells in vivo, we sensitized mice with SEB mixed with alum (an immune adjuvant). The sensitized mice were then gavage-fed with SEB (0.1 mg/mouse) daily for 3 days. After sacrifice, the expression of TNFAIP3 was significantly increased in sensitized mice (Fig.3C).

### Inhibition of TNFAIP3 Results in SEB Transport Across Polarized Enterocytes

**Figure 4:**
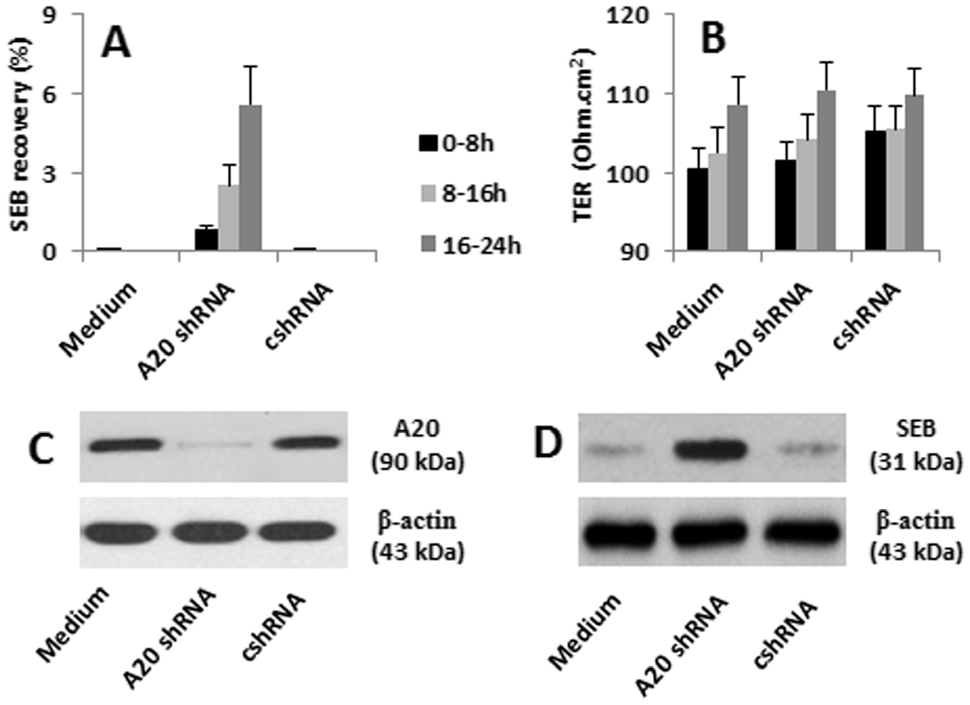
TNFAIP3 prevents SEB transport across HT-29 monolayers. HT-29 cells were cultured in transwells to confluence. SEB was added to the apical chambers at a concentration of 10 µg/ml. Medium samples were taken from the basal chambers at 8 h, 16 h and 24 h respectively. SEB in the samples was assessed by ELISA. A, the bars indicate the SEB contents. The Y axis indicates the SEB recovery (%SEB contents in the apical chambers). B, the bars indicate the TER increase rate of the HT-29 monolayers after exposure to SEB for 24 h. The data were presented as mean ± SD in A and B. C, the immune blots show the TNFAIP3 knockdown results. D, the cells were collected at 24 h time point; the cell extracts were analysed by Western blotting. The immune blots show the SEB retained in the enterocytes. The data represent three separate experiments.

To elucidate whether the endocytic SEB was degraded within the cells or transported to the transwell basal chambers, SEB-specific IgE primed enterocytes were cultured to confluence in transwells. SEB was added to the apical chambers; the culture media were sampled from the basal chambers. As analyzed by ELISA, within the 24 h incubation, no detectable SEB was observed in the basal chambers (Fig.4A). The results indicate that under physiological condition, enterocytes can endocytose SEB that can be degraded within the cells.

Based on the results, the expression of TNFAIP3 was increased in enterocytes upon the absorption of SEB, no detectable SEB was observed in the basal chambers, and we postulated that TNFAIP3 facilitated the degradation of the endocytic SEB in enterocytes. To test the hypothesis, we knocked down the TNFAIP3 gene in enterocytes; the cells were used in experiments with the same experimental procedures. The results showed that abundant SEB was converted to the basal chambers (Fig.4A). The data implicate that TNFAIP3 can facilitate the degradation of SEB in enterocytes.

To elucidate if the SEB in basal chambers was transported through the paracellular spaces or via the intracellular pathway, we recorded the TER (the indicator of the opening of the paracellular spaces) of HT-29 monolayers. After the exposure to SEB in culture for 24 h, the TER of HT-29 monolayers was increased about 10% in both TNFAIP3-sufficient and TNFAIP3-deficient HT-29 monolayers (Fig.4B). The gene silence of TNFAIP3 in HT-29 cells was presented in Fig.4C. The results imply that SEB does not alter the paracellular permeability of HT-29 monolayers; the SEB in the basal chambers was transported via the intracellular pathway. To see if knockdown of the TNFAIP3 gene could result in the the retain of SEB within the enterocytes, we assessed the SEB levels in the enterocytes. Indeed, the SEB levels were significantly higher in the enterocytes with TNFAIP3-deficiency than those TNFAIP3-sufficient enterocytes (Fig.4D).

### TNFAIP3 is Required in SEB-carrying Endosomes Fuse with Lysosomes

**Figure 5:**
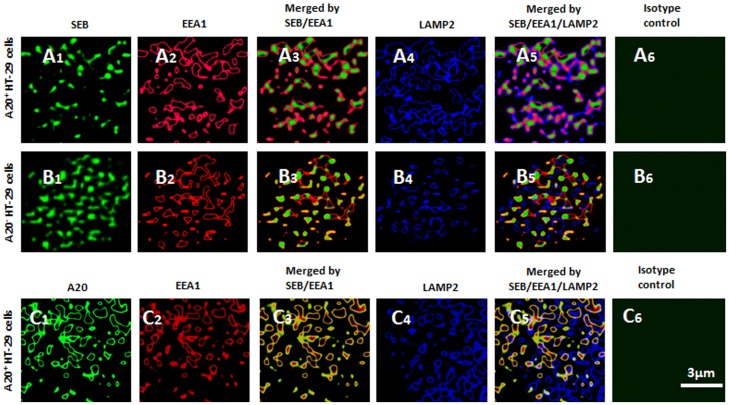
Immune staining of SEB (or TNFAIP3)/EEA1/LAMP2 in HT-29 cells. SEB-specific IgE primed HT-29 cells were exposed to SEB in culture for 24 h. The cells were analyzed by immune staining. The representative confocal images show the split images that include positive staining of SEB (A1, B1; in green), EEA1 (A2, B2, C2; in red), LAMP2 (A4, B4, C4; in blue) and TNFAIP3 (C1; in green), which were also annotated above each panel (Each panel shows the image of one single HT-29 cell). The image analysis data of cells were presented in Table 1. Thirty cells were analyzed for each group. Original magnification: ×630.

Previous reports indicate that TNFAIP3 plays a role in the tethering of endosomes to lysosomes [7]. To further understand the mechanism by which TNFAIP3 facilitates the degradation of SEB in enterocytes, we observed the role of TNFAIP3 in the fusion of SEB-carrying endosome and lysosome in HT-29 cells. The cells were primed by SEB-specific IgE and then exposed to SEB in culture for 24 h. By immunocytochemistry, colocalizations of SEB/EEA1/LAMP2 immune positive products were observed in SEB-treated HT-29 cells (Fig.5A). In TNFAIP3 deficient enterocytes, less SEB/EEA1/LAMP2 immune positive products, but more SEB/EEA1 positive staining were observed (Fig.5B). The results imply that TNFAIP3 can facilitate the fusion of SEB-carrying endosome and lysosome in enterocytes. To test the inference, SEB-treated HT-29 cells were stained with antibodies of TNFAIP3, LAMP2 and EEA1. The confocal microscopy results showed that in TNFAIP3 sufficient HT-29 cells, the immune positive products of TNFAIP3/LAMP2/EEA1, or TNFAIP3/EEA1 were observed (Fig.5C). The results indicate that upon absorption of SEB, enterocytes express TNFAIP3 that is localized in the endosomes. TNFAIP3 tethers SEB-carrying endosomes to lysosomes to facilitate the fusion of endosome and lysosome.

**Table 1:** Measurements of SEB/EEA1/LAMP2 staining in HT29 cells.

Immune staining	Cells	Particles	SEB^+^	SEB^+^/EEA1^+^	SEB^+^/EEA1^+^/LAMP2^+^
**TNFAIP3^+^ HT29 cell**	30	1147	100%	80.09 (9.8)%	75.26 (8.6)%
**TNFAIP3^–^ HT29 cell**	30	1185	100%	*96.69 (11.5)%	*4.52 (5.3)%
	Cells	Particles	**TNFAIP3**	**TNFAIP3/EEA1**	**TNFAIP3/EEA1/LAMP2**
**TNFAIP3^+^ HT29 cell**	30	1227	100%	97.8 (9.1)%	91.2 (9.9)%

The immune positively stained particles in Fig.5 were counted in 30 cells. The data were presented as mean (SD). *, p<0.01, compared with TNFAIP3^+^ group.

## Discussion

The nutrients in the gut lumen are mixed with many other substances including microbial products. How the gut epithelial cells differentially absorb nutrients from other noxious substances has not been fully understood yet. The present data have revealed that the gut epithelial cells not only can absorb nutrients, but also can absorb SEB, which can be degraded within the epithelial cells before delivering to the basal sides (similar to the subepithelial region in the intestine). Upon the uptake of SEB, the epithelial cells express TNFAIP3, which facilitates the fusion of endosome and lysosome.

SEB is produced by *S. aureus*; the latter is a common opportunity pathogen. SEB can be detected in nasal discharge of patients with chronic sinusitis, which may be swallowed down into the gastrointestinal tract, that was demonstrated by our previous study that SEB was involved in the pathogenesis of intestinal chronic inflammation in patients with both chronic sinusitis and inflammatory bowel disease; the patients also had high serum levels of SEB-specific IgE [18]. The association between SEB and intestinal inflammation was demonstrated by animal models as well [13], [19]. The increase in SEB-specific IgE in patients with allergic diseases was reported by a number of investigators [20], [21]. Published data indicate that gut epithelial cells express CD23, the low affinity receptor of IgE [22], that can convert the endogenous antigen specific IgE to the surface of enterocytes to form a complex of IgE/CD23 [23]. The present data are in line with the reports by showing the immune complex of SEB/IgE/CD23 on the surface of the enterocytes. Further evidence from the present study indicates that the immune complex is formed in the absorption of SEB by the enterocytes; the absorption of SEB is much more in the enterocytes pre-sensitized by SEB-specific IgE than that in naïve enterocytes. The phenomenon implies that naïve enterocytes can absorb microbial products such as SEB that can be significantly enhanced in sensitized enterocytes as well as the sensitized mice. Further results indicate that the removal of CD23 from the surface of the HT-29 cells; the fact adds supportive evidence that CD23 is involved in mediating the absorption of SEB in the cells pre-sensitized by SEB-specific IgE. Interestingly, we noted that after primed with SEB-specific IgE and exposure to SEB, more CD23 expression was detected in the enterocytes. The phenomenon implies that exposure to either IgE or the specific antigen or both of them may up regulate the expression of CD23 on target cells, which needs to be further investigated.

From the complexes of SEB/IgE/CD23 to endosome formation in the cytoplasm, there may be more biochemistry reactions in the cell membrane. We noticed the phenomenon in an allergy rat model. In an ex vivo experiment, we saw the sensitized rat tracheal epithelial cells “ate” antigens (depicted by an electron photomicrograph); the antigens were wrapped by the plasma membrane to form endosomes in the cytoplasm [24]. Lately, we observed the intestinal epithelial cells expressed CD23 on the cell surface that facilitated the “rapid transport of antigen across the epithelial barrier” [22], [23], [25]. By studying the proteinase-activated receptor-2-mediated antigen transport across the epithelial barrier, we noted a series of signal transduction pathway components, such as ERK1/2, beta-arrestin, were involved in the receptor activation [26]. Thus, between the SEB/IgE/CD23 internalization in enterocytes and the production of TNFAIP3, there may be a cascade of biochemical reactions in the enterocytes, which need further investigation.

Cumulative reports indicate that TNFAIP3 plays a critical role in maintaining the homeostasis in the body via suppressing the NF-κB. Short of TNFAIP3 results in a number of diseases, such as TNFAIP3-deficient B cells prone to producing auto antibodies to contribute to autoimmune diseases [27], [28]. Knocking out TNFAIP3 gene from the intestinal epithelial cells induces severe inflammation in the intestine [8]. Our results are in line with the data by showing a gut epithelial cell line, the HT-29 cell, expressed TNFAIP3. The results also show that, upon the exposure to SEB in enterocytes, the expression of TNFAIP3 was markedly increased. The results are in line with a previous study, in which Hishamatsu et al noted that, the exposure to microbial product, peptidoglycan, increased the expression of TNFAIP3 in macrophages [29]. Such a phenomenon is interpreted as that the increase in TNFAIP3 in the cell is to conquer the increase in the proinflammatory cytokine transcription factor, NF-κB [29]. Inspired by this, we infer that the increase in TNFAIP3 in HT-29 cells upon the absorption of SEB is to facilitate the degradation of SEB in enterocytes. The inference is corroborated by the subsequent data that TNFAIP3 is indeed involved in the degradation of the absorbed SEB in enterocytes. The data demonstrate that TNFAIP3 not only has immune regulatory function [8], [27], [28], but is also involved in the protein degradation in the gut epithelium.

The present data indicate that the gut epithelial cells not only absorb the necessary nutrients, but also absorb microbial products such as SEB. SEB is an exotoxin from a pathogenic bacterium, *S. aureus*, and can directly activate T cells and induce shock [30], [31]. However, SEB does not compromise the gut epithelial barrier function directly as we observed previously [16], [32]. The present data are in line with the data [16], [32] and show that exposure to SEB do not alter the TER of HT-29 monolayer as well as little SEB was transported across the TNFAIP3-sufficient HT-29 monolayers. The data imply that the absorbed SEB probably degraded within the HT-29 cells.

The endocytosis is a common mechanism by which cells absorb exogenous substances. Our data show that after exposure to SEB, the SEB-carrying endosomes were localized inside of HT-29 cells; the finding indicates that the endocytosis is employed by HT-29 cells to absorb SEB. The endosome/lysosome fusion is the subsequent step by which cells digest the internalized materials by the proteolytic enzymes in the lysosomes [4]. Further evidence shows that the complexes of SEB, endosome marker (EEA1) and lysosome marker (LAMP2) were colocalized in the naïve HT-29 cells indicating the fusion of SEB-carrying endosomes and lysosomes. A novel phenomenon was noted in the present study; in TNFAIP3-deficient HT-29 cells, a large number of SEB-carrying endosomes were observed in HT-29 cells, but few SEB^+^ endosome/lysosome were observed; the phenomenon indicates the lacking TNFAIP3 prevents the SEB-carrying endosome from fusing the lysosome in the HT-29 cells since TNFAIP3 can tether endosomes to lysosomes [7]; or in other words, TNFAIP3 is required in the endosome/lysosome fusion. Further supportive data were obtained that in TNFAIP3-deficient HT-29 cells, few endosome/lysosome fusions were observed.

Taken together, the present study reveals that the human gut epithelial cell line, HT-29 cell, can absorb SEB via binding to IgE/CD23 complexes on the cells; the absorbed SEB can be digested within the cells; TNFAIP3 plays an important role in the degradation of the endocytic SEB in HT-29 cells by promoting the endosome/lysosome fusion.
